# Aryl hydrocarbon receptor/cytochrome P450 1A1 pathway mediates breast cancer stem cells expansion through PTEN inhibition and β-Catenin and Akt activation

**DOI:** 10.1186/s12943-016-0570-y

**Published:** 2017-01-19

**Authors:** Abdullah Al-Dhfyan, Ali Alhoshani, Hesham M. Korashy

**Affiliations:** 1 Department of Pharmacology and Toxicology, College of Pharmacy, King Saud University, P.O. Box 2457, Riyadh, 11451 Saudi Arabia; 2 Stem Cell & Tissue Re-Engineering, King Faisal Specialist Hospital and Research Center, Riyadh, 11211 Saudi Arabia

**Keywords:** AhR, CYP1A1, Cancer stem cells, Breast cancer, β-Catenin, PI3K/Akt, PTEN, TCDD, shRNA, Balb/c mice

## Abstract

**Background:**

Breast cancer stem cells (CSCs) are small sub-type of the whole cancer cells that drive tumor initiation, progression and metastasis. Recent studies have demonstrated a role for the aryl hydrocarbon receptor (AhR)/cytochrome P4501A1 pathway in CSCs expansion. However, the exact molecular mechanisms remain unclear.

**Methods:**

The current study was designed to a) determine the effect of AhR activation and inhibition on breast CSCs development, maintenance, self-renewal, and chemoresistance at the in vitro and in vivo levels and b) explore the role of β-Catenin, PI3K/Akt, and PTEN signaling pathways. To test this hypothesis, CSC characteristics of five human breast cancer cells; SKBR-3, MCF-7, and MDA-MB231, HS587T, and T47D treated with AhR activators or inhibitor were determined using Aldefluor assay, side population, and mammosphere formation. The mRNA, protein expression, cellular content and localization of the target genes were determined by RT-PCR, Western blot analysis, and Immunofluorescence, respectively. At the in vivo level, female Balb/c mice were treated with AhR/CYP1A1 inducer and histopathology changes and Immunohistochemistry examination for target proteins were determined.

**Results:**

The constitutive mRNA expression and cellular content of CYP1A1 and CYP1B1, AhR-regulated genes, were markedly higher in CSCs more than differentiating non-CSCs of five different human breast cancer cells. Activation of AhR/CYP1A1 in MCF-7 cells by TCDD and DMBA, strong AhR activators, significantly increased CSC-specific markers, mammosphere formation, aldehyde dehydrogenase (ALDH) activity, and percentage of side population (SP) cells, whereas inactivation of AhR/CYP1A1 using chemical inhibitor, α-naphthoflavone (α-NF), or by genetic shRNA knockdown, significantly inhibited the upregulation of ALDH activity and SP cells. Importantly, inactivation of the AhR/CYP1A1 significantly increased sensitization of CSCs to the chemotherapeutic agent doxorubicin. Mechanistically, Induction of AhR/CYP1A1 by TCDD and DMBA was associated with significant increase in β-Catenin mRNA and protein expression, nuclear translocation and its downstream target Cyclin D1, whereas AhR or CYP1A1 knockdown using shRNA dramatically inhibited β-Catenin cellular content and nuclear translocation. This was associated with significant inhibition of PTEN and induction of total and phosphorylated Akt protein expressions. Importantly, inhibition of PI3K/Akt pathway by LY294002 completely blocked the TCDD-induced SP cells expansion. In vivo, IHC staining of mammary gland structures of untreated and DMBA (30 mg/kg, IP)- treated mice, showed tremendous inhibition of PTEN expression accompanied with an increase in the expression p-Akt, β-Catenin and stem cells marker ALDH1.

**Conclusions:**

The present study provides the first evidence that AhR/CYP1A1 signaling pathway is controlling breast CSCs proliferation, development, self-renewal and chemoresistance through inhibition of the PTEN and activation of β-Catenin and Akt pathways.

## Background

The hypothesis that tumors are organized in a cellular hierarchy driven by cancer stem cells (CSCs) has fundamental implications for oncology and clinical implications for the early detection, prevention, and treatment of cancer [1]. CSCs are small sub-type of the whole cancer cells that drive tumor initiation, progression and metastasis. CSCs hypothesis postulates that tumors are maintained by a self-renewing CSC population that is also capable of differentiating into non-self-renewing cell populations which constitute the bulk of the tumor [2], and thus are considered as novel therapeutic targets for cancer treatment and/or prevention. For example, as few as 200 of CSCs can generate tumors in human non-obese diabetic-severe combined immune deficiency (NOD/SCID) mice whereas 20,000 cells that did not display this phenotype failed to generate tumor [3]. CSCs have been identified in leukemia [4], breast [3], brain [5], lung [6], colon [7] and other cancer types.

CSCs are characterized by the capacity to form tumor spheres (mammospheres), expression of high levels of ATP-binding cassette (ABC) drug transporters (particularly ABCG2), which all collectively results in resistance to chemotherapies and hence recurrence and ultimately death because of treatment failure [8, 9]. Breast CSCs can be identified and isolated by fluorescence-activated cell sorting (FACS) of aldehyde dehydrogenase-1 (ALDH) [10] and by a side population (SP) phenotype. In breast tumors, the use of neoadjuvant regimens showed that conventional chemotherapy could lead to enrichment in breast CSCs in treated patients as well as in xenografted mice [11]. This suggests that many cancer therapies, while killing the bulk of tumor cells, may ultimately fail because they do not eliminate breast CSCs, and thus regenerate new tumors.

CSCs biology such as development and maintenance is controlled by several signaling pathways such as Wnt and Notch pathways. Mechanistically, Wnt pathway is known to mediate CSC self-renewal through modulation of β-Catenin/TCF transcription factor, whereas CSC maintenance and differentiation are governed by Notch/Hes pathway [12]. In addition, it has been shown that phosphatidylinositol 3-kinase (PI3K)/Akt signaling pathway plays a role in the regulation of several genes in carcinogenesis such as β-Catenin, p21, p27, Mdm2 or Forkhead transcription factors [13]. Importantly, these CSC-regulating pathways have been shown to crosstalk with several transcription factors and signaling pathways such as the aryl hydrocarbon receptor (AhR) [14].

The Aryl hydrocarbon receptor (AhR) is a cytosolic ligand-activated transcriptional factor that is involved in the regulation of cell differentiation, proliferation, and cancer imitation [15, 16]. Activation of AhR upon binding to its ligand, such as 2,3,7,8-tetrachlorodibenzo[*p*]dioxin (TCDD) or 7,12-dimethybenz[a]anthracene (DMBA), initiates the transcriptional regulation of a number of genes such as the cytochrome P450 1A1 (CYP1A1) and CYP1B1 [17, 18]. Induction of CYP1A1 and CYP1B1 is known to bioactivate environmental toxicants into their ultimate carcinogenic epoxide and diol-epoxide intermediates [19], which can bind covalently to DNA forming DNA adducts and subsequent tumor initiation. The carcinogenic role of AhR/CYP1 family is supported by the fact that DMBA, a powerful breast-specific laboratory carcinogen, induces cancer in wild type, but not in cyp1 knockout, mice [20]. In addition, recent studies from our laboratory and others have demonstrated the pivotal role of AhR/CYP1A1 in development of breast cancer. In that, it has been previously reported that TCDD, AhR ligand and potent CYP1A1 inducer, increased human breast cancer MCF-7 cell proliferation and the number and size of the mammospheres (CSC populations) [21] via the suppression of apoptosis [22]. In addition, Maayah et al., have demonstrated that inhibition of AhR/CYP1A1 pathway protects against breast cancer initiation and adduct formation in human epithelial breast MCF10A cells [23].

Since CSCs, as tumor-initiating cells, are major targets for chemical carcinogens, it is highly possible that AhR/CYP1A1 signaling pathway plays a role in controlling CSCs. However, the exact role and molecular mechanism of the AhR/CYP1A1 signaling pathway in developing, maintaining, and controlling CSCs remains fully uninvestigated. Thus, the current study was designed to a) examine the functional relevance of constitutive and inducible AhR/CYP1A1 expression on breast CSC properties, proliferation, development, maintenance and self-renewal and b) verify the molecular mechanisms mediating AhR/CYP1A1 effects on CSCs, particularly the role of Wnt/β-Catenin, Notch/Hes, and PTEN-PI3K/Akt pathways.

## Methods

### Materials

DMBA, TCDD, and alpha-naphthoflavone (α-NF) were purchased from Toronto Laboratories Research (Toronto, Canada). Dulbecco’s Modified Eagle’s Medium (DMEM), TRIzol reagent, Annexin V-FITC/Propidium Iodide (PI) were purchased from Invitrogen Co. (Grand Island, NY). FLI 06, LY294002, sodium azide, and puromycin were purchased from Sigma-Aldrich Co., (St. Louis, MO), whereas XAV-939 was obtained from Tocris Bioscience (Bristol, UK). High Capacity cDNA Reverse Transcription kit and SYBR® Green PCR Master Mix were purchased from Applied Biosystems® (Foster city, CA). DNA primers were obtained from Integrated DNA Technologies (Coralville, IA). Aldefluor^®^ kit was purchased from Stem Cell Technologies (Vancouver, CANADA). Acrylamide/bisacrylamide 30% (29:1) and nitrocellulose membrane were purchased from Bio-Rad Laboratories (Hercules, CA). Vybrant apoptosis assay kit Molecular Probes® were obtained from Thermo Fisher Scientific (Waltham, MA). Primary and Horse Radish Peroxidase (HRP)-conjugated secondary antibodies against target proteins, AhR and CYP1A1 shRNA, and transfection reagent systems were ordered from Santa Cruz Biotechnology, Inc. (Santa Cruz, CA). The Western blot detection kits, Luminata® Western HRP Chemiluminescence Substrates were purchased from EMD Millipore (Billerica, MA).

### Breast cancer cell culture model

Human breast cancer cell lines; SKBR-3 (human epidermal growth factor receptor 2 +, HER2 +), MCF-7 (estrogen receptor+, ER+), T47D (ER+), and triple negative breast cancer (TNBC) cells (ER-, HER2-, and progesterone receptor-); MDA-MB-231 and HS578T were obtained from the American Type Culture Collection (Rockville, MD). The cells were cultivated in DMEM with phenol red, 10% fetal bovine serum, 200 μM L-glutamine, 1X Antibiotic-Antimycotic and grown in 75 or 150 cm^2^ tissue culture flasks at 37 °C under a 5% CO_2_ humidified environment.

The cells were seeded in cell culture plates in DMEM culture media for RNA, protein assays, and flow cytometry. In all experiments, the cells were treated for the indicated time intervals in complete media. Stock solutions of all utilized chemicals were prepared fresh in dimethyl sulfoxide (DMSO) just prior each experiment, where the DMSO concentration in all treatment did not exceed 0.1% (v/v).

### Mammosphere formation assay

Breast CSCs can be enriched by culturing of cells in non-adherent non-differentiating conditions. Confluent MCF-7 cells were first harvested and counted using trypan blue to exclude the dead cells. Thereafter, live cells after treatment either with control media (0.1% DMSO) or media containing different concentration of TCDD or DMBA in the presence and absence of alpha-naphthoflavone (α-NF) for three days were suspended at 1000 cells/100 μL and seeded into ultralow attachment plates (Corning, NY, USA) in serum-free mammary epithelium basal medium, supplemented with B27 1:50, 20 ng/mL epidermal growth factor (EGF), 5 μg/mL insulin, 1% ABM, and 0.5 μg/ml hydrocortisone as described previously [24, 25]. Cells grown in these conditions as non-adherent spherical clusters (mammospheres) were allowed to grow for 7 days. Thereafter, the mammosphere cell numbers were determined by Evos® transmitted light microscope, Life Technologies Co., (Grand Island, NY) [24, 25]. Equal numbers of mammosphere (CSCs) and adherent (non-CSCs) cells were collected for the mRNA, ALDH, and SP experiments.

### RNA isolation and quantification of the mRNA expression by Real Time-PCR (RT-PCR)

Total RNA from MCF-7 cells was isolated using TRIzol reagent according to manufacturing instructions (Invetrogen^®^). RNA quality and purity were determined spectrophotometrically by measuring the 260/280-absorbance ratio maintained in the range of ~ 2.0 optical density. Human primers for AhR (F: ACATCACCTACGCCAGTCGC and R: TCTATGCCGCTTGGAAGGAT), CYP1A1 (F: CTATCTGGGCTGTGG GCAA and R: CTGGCTCAAGCACAACTTGG), CYP1B1 (F: AACCGCAACTTCAGCAACTT and R: GAGGATAAAGGCGTCCATCA), and β-ACTIN (F: TATTGGCAACGAGCGGTTCC and R: GGCATAGAGGTCTTTACGGATGTC) were synthesized and obtained from Integrated DNA Technologies (Coralville, IA). Quantitative mRNA expression of AhR, CYP1A1, and CYP1B1 were quantified using ABI QuantStudio® 6 Flex fast RT-PCR System, Life Technologies Co., (Grand Island, NY) as described previously [26]. The fold change in the level of target mRNAs between treated and untreated cells were corrected by the level of β-ACTIN. The RT-PCR data were analyzed using the relative gene expression (i.e.,ΔΔ CT) method as described previously [27] using the following equation: fold change = 2^− Δ(ΔCt)^, where ΔCt = Ct_(target)_ − Ct_(β-actin)_ and Δ(ΔCt) = ΔCt_(treated)_ − ΔCt_(untreated)_.

### Immunofluorescence assay

Immunofluorescence assay was conducted as described previously [28]. Briefly, MCF-7 cells treated with the test compounds for the indicated time intervals were plated on glass slides at a density of 20000 cells/cm^2^ and cultured for 7 days then fixed in 4% formaldehyde. Fixed cells were then stained with primary antibodies against ALDH, CYP1A1, or β-Catenin, followed by fluorescence isothiocyanate (FITC) conjugated secondary antibodies and 1 μg/mL 4′-6-Diamidino-2-phenylindole (DAPI), a fluorescent stain for nuclear DNA. Each sample was stained in triplicate for each antibody. The fluorescence staining intensity and intracellular localization were then examined by BD Pathway 855 Bioimager (San Jose, CA).

### Aldeluor assay

Aldeflour^®^ assay from Stem Cell Technologies (Vancouver, Canada) is a novel fluorescent reagent system used for the identification, evaluation, and isolation of stem cells based on the enzymatic activity of ALDH [10]. After treatment with tests compounds for 72 h, MCF-7 cells were harvested, washed and incubated in Aldefluor assay buffer containing an ALDH substrate (BAAA) with or without the addition of diethylaminobenzaldehye (DEAB), an ALDH inhibitor. Thereafter, percentage of ALDH+ cells were determined by flow cytometry.

### Side population (SP)

SP analysis is a valuable technique for identifying and sorting CSCs in a variety of tissues and species. MCF-7 cells treated with test compounds for 72 h were harvested, pelleted, and then suspended in DMEM cell culture medium. The cells were then incubated with Dye Cycle Violet (DCV), a cell-permeable DNA binding dye, at a final staining concentration of 10 μM. Propidium iodide (PI) stain were utilized to exclude dead cells. The ability of CSCs to exclude DCV dye, appeared as a distinct dim ‘tail’ in the flow cytometry plot, was determined using LSRII flow cytometer, BD Biosciences (San Jose, CA). For SP cells sorting, identification and isolation of SP and non-SP MCF-7 cells were performed by Fluorescence-Activated Cell Sortingthe BD FACSAria® flow cytometer cell sorter, BD Biosciences, (San Jose, CA). Debris and cell clusters were excluded by side-scatter and forward-scatter analyses.

### Protein extraction and Western blot analysis

Total protein from MCF-7 cell lysate was extracted as described previously [29] and the protein concentrations were quantified using Direct Detect® Infrared Spectrometer, EMD Millipore Co. (Billerica, MA). Western blot analysis was performed as described before [29] with slight modifications. Briefly, 30–60 μg of protein from each treatment group was separated by 10% sodium dodecyl sulfate (SDS)-polyacrylamide gel electrophoresis (PAGE), and then electrophoretically transferred to nitrocellulose membrane. Using SNAP i.d.® 2.0 Protein Detection System, EMD Millipore (Billerica, MA). Protein membranes were incubated with blocking solution for 10 min, followed by primary antibodies against target proteins for 5 min and then with peroxidase-conjugated IgG secondary antibodies for 10 min at room temperature, as per manufacturer’s instructions. The protein bands were detected using Luminata® Western HRP Chemiluminescence Substrates and then visualized and quantified by C-DiGit® Blot Scanner, LI-COR Biosciences (Lincoln, NE).

### Apoptosis assay

The percentage of cells undergoing to apoptosis as indicator of response to chemotherapy was determined using Vybrant apoptosis assay kit (Annexin V, APC conjugate; Molecular Probes^®^) by flow cytometer [30]. For this purpose, SP and non-SP MCF-7 cells treated with test compounds for 72 h were harvested, pelleted, and then suspended in DMEM cell culture medium. Thereafter, a total of 10^4^ cells were collected and then stained with annexin V and DAPI, as a viability dye, and immediately analyzed on the BD LSRII Flow Cytometer (San Jose, CA). Cells were considered viable if they are double negative for Annexin V and DAPI.

### Gene silencing using shRNA RNA interference and lentiviral transfection

MFC-7 cells were seeded in 24-well cell culture plates at a density of 5 × 10^4^ cells/well and allowed to grow overnight. Cells were then transfected with appropriate concentrated lentivirus AhR and CYP1A1 shRNA or their control shRNA as per manufacturer’s instructions, Santa Cruz Biotechnology, Inc. (Santa Cruz, CA). The stably transfected MCF-7 cells were selected with puromycin (2 μg/ml). Once reached confluence, the cells were trypsinized and cultured in 6-well cell culture plates and then were treated for 72 h with 0.1% DMSO, TCDD 10 nM or DMBA 5 μM.

### Animals and experimental design

Six weeks-old virgin female Balb/c mice, weighing 20–25 g, obtained from the Animal Care Center, College of Pharmacy, King Saud University, Riyadh, Saudi Arabia, were housed in cages under controlled environmental conditions (25 °C and a 12 h light/dark cycle). Mice were randomly divided into two groups of 10 mice each as follows: Group I: received vehicle corn oil IP and served as Control, whereas group II received a single dose of DMBA (30 mg/kg, IP) and served as DMBA-treated. All animals had free access to pulverized standard rat pellet diet. The protocol of this study has been approved by Research Ethics Committee of College of Pharmacy, King Saud University. All animals were handled in accordance with guide for the Care and Use of Laboratory Animals by National Institute of Health. After two months, all animals were decapitated and immediately mammary fat pad glands were excised and divided into two sections for histopathology and immunohistochemistry (IHC) assays.

### Histopathology

Three micron thick sections from mammary glands of both untreated and DMBA-treated mice were stained with routine hematoxylin and eosin stains (H&E) as described before [31]. The stained sections were viewed and studied using Zeiss Axiovert 40 CFL microscope, Carl Zeiss LLC (Thornwood, NY) by expert histopathologists. The adequacy of the sample in each case was checked on the semi-thin sections. The thin sections were stained with uranyl acetate and lead citrate then all the sections were examined and photographed by the same histopathologist.

### Immunohistochemistry (IHC) assay

IHC were performed on formalin-fixed, paraffin-embedded sections from mammary glands of both untreated and DMBA-treated mice as described previously [32]. Tissue sections were incubated overnight at 4 °C with the following primary antibodies diluted in PBS- 0.1% BSA: anti-CYP1A1 (1:50); anti-ALDH (1:50); anti-PTEN (1:50); anti-p-Akt (1:50); and anti-β-Catenin (1:50). After washing, sections were stained for 30 min at RT, with Labeled Polymer (EnVision+) HRP detection kit as a secondary antibody. Color was developed with 3,3′-diaminobenzidine (DAB) and instant hematoxylin was used for counterstaining. Slides were observed under Zeiss A xiovert 40 CFL microscope, Carl Zeiss LLC (Thornwood, NY).

### Statistical analysis

All results were presented as mean ± SEM. The comparative analyses of the results from various experimental groups with their corresponding controls were performed using SigmaStat^®^ for Windows, Systat Software Inc., (San Jose, CA). Student’s *t*-test or one-way analysis of variance (ANOVA) followed by Student–Newman–Keul’s test was carried out to assess which treatment groups show significant differences from the control ones. The differences were considered significant when *p* < 0.05.

## Results

### Constitutive expression of CYP1A1 and CYP1B1 in CSCs vs. non-CSCs populations of different breast cancer cell lines

**Fig. 1:**
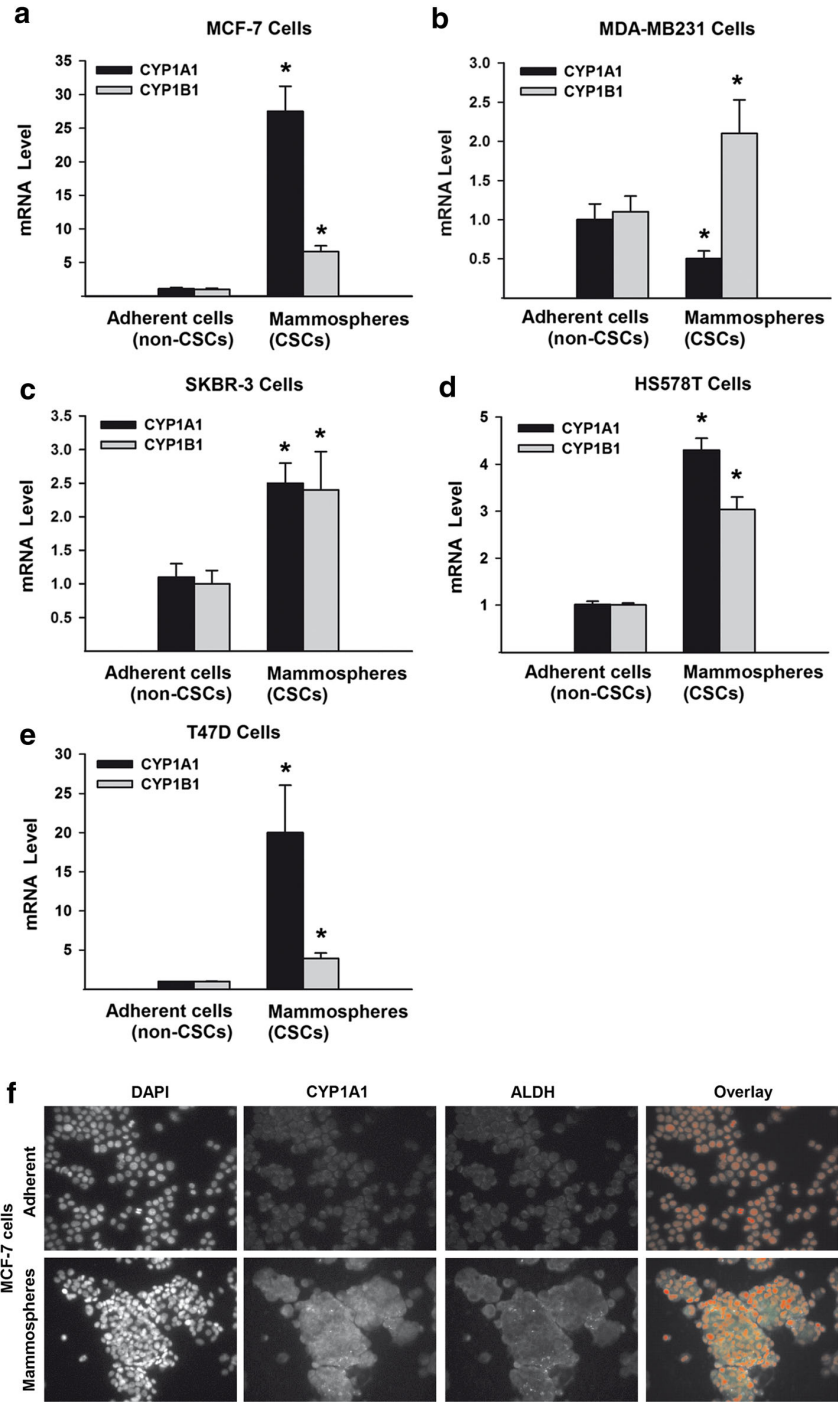
Constitutive expression of CYP1A1 and CYP1B1 in mammospheres vs adherent breast cancer cell lines. The basal mRNA expression of CYP1A1 and CYP1B1 of human breast cancer cell lines; MCF-7 (**a**), MDA-MB231 (**b**), SKBR-3 (**c**), HS578T (**d**), and T47D (**e**) were determined by RT-PCR normalized to β-ACTIN housekeeping gene. Duplicate reactions were performed for each experiment and the values are presented as mean ± SEM (*n* = 6). *; *p* < 0.05 compared to corresponding adherent cells. **f** Mammosphere and adherent populations of MCF-7 cells were stained with primary antibodies against CYP1A1 (*green*) and ALDH (*magenta*) followed by secondary antibodies and DAPI (*red*). Thereafter, the constitute CYP1A1 and ALDH proteins localization was conducted by Immunofluorescence assay. Each sample was stained in triplicate for each antibody

To examine the constitutive level of CYP1A1 and CYP1B1 expression in stem/progenitor cells and its counterpart non-CSCs cells, we quantified the mRNA expression levels of CYP1A1 and CYP1B1 in the mammosphere (CSCs) and adherent (non-CSCs) populations of five different breast cancer cell lines that represent different hormonal heterogeneity. These cells include SKBR-3 (HER-2 +), MCF-7 and T47D (ER +), MDA-MB 231 and HS578T (triple negative). Figure 1a-e shows that generally the basal expressions of CYP1A1 and CYP1B1 mRNA levels were higher in mammospheres (CSCs) than in adherent cells (non-CSCs). In particular, the mammosphere (CSCs) of all cancer cells tested (Fig. 1a, c-e), with the exception of MDA-MB231 (Fig. 1b), showed higher CYP1A1 mRNA levels than CYP1B1. Interestingly, MCF-7 mammosphere cells showed the highest expression levels of CYP1A1 (27-fold) and CYP1B1 (7-fold) than corresponding adherent cells among all cancer cells (Fig. 1a), followed by T47D (20-fold for CYP1A1 and 4-fold for CYP1B1) (Fig. 1e). The CYP1A1 and CYP1B1 mRNA expression levels in SKBR-3 and HS587T cells showed higer levels in their mammosphere than adherent cells by approximately 2.5- and 4-fold, respectively (Fig. 1c and d). On the other hand, although the expression of CYP1A1 mRNA was slightly decreased in MDA-MB231 mammospheres as compared to their adherent cells, CYP1B1 mRNA level, which is another marker for AhR activity, was significantly increased by 2-fold (Fig. 1b). According to these results, human breast cancer MCF-7 cell line, that showed the highest expression levels, was utilized as a study model for investigating the role of AhR/CYP1A1 pathway in regulating and controlling CSCs.

To further confirm the overexpression of CYP1A1 in MCF-7 CSCs, CYP1A1 content and cellular localization in MCF-7 mammospheres vs adherent were determined using immunofluorescence assay. As shown in Fig. 1f, CYP1A1 cellular content and localization (green) was overexpressed in mammosphere more than in adherent MCF-7 cells. Importantly, the fluorescence signals of CYP1A1 immunoreactivity completely coincided with increased CSCs specific marker, ALDH fluorescence content and cellular localization (magenta), a well-known CSC-specific marker.

### Effects of AhR activation and CYP1A1 induction on MCF-7 mammosphere formation

**Fig. 2:**
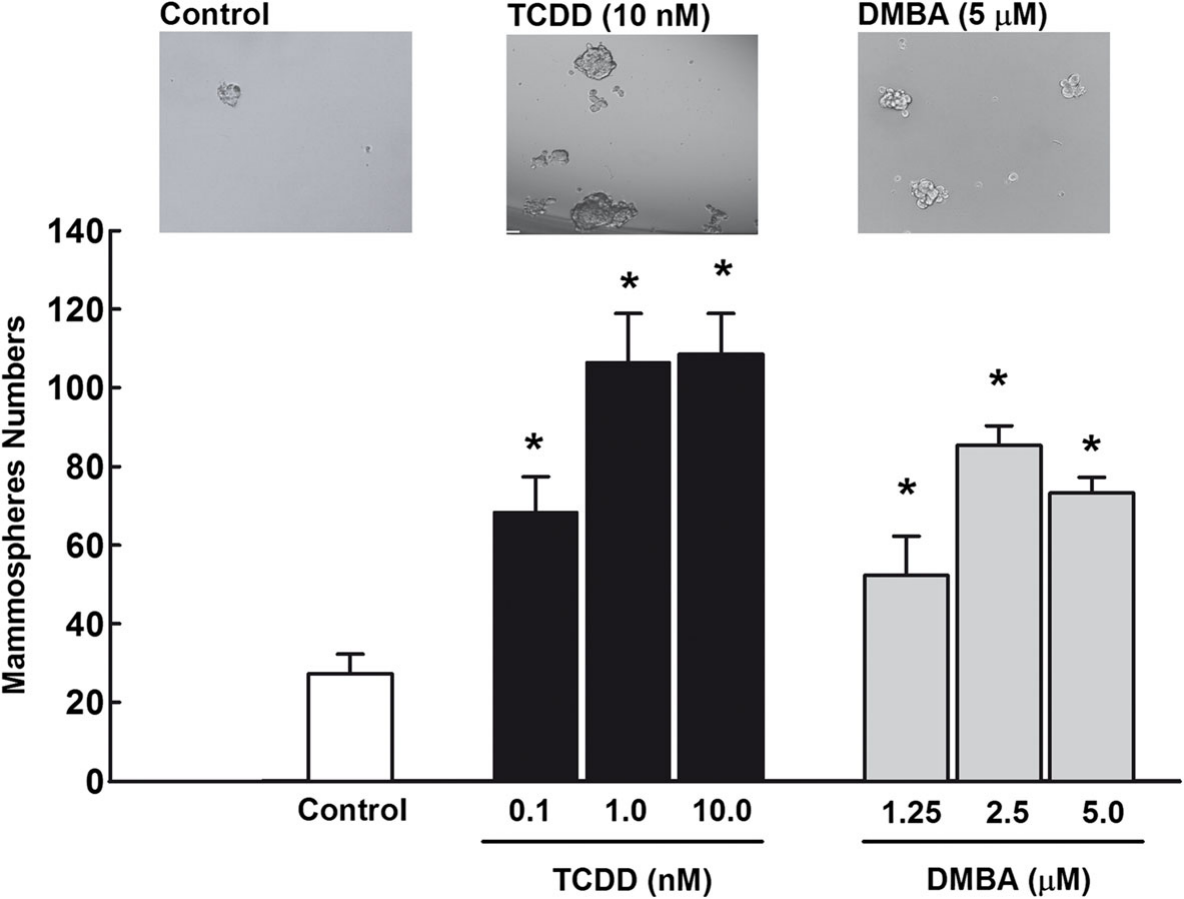
Effects of AhR activation on breast cancer mammospheres formation. MCF-7 cells were trated with indicated concentrations of TCDD and DMBA for 72 h. Thereafter, cells were trypsinized and viable 1000 cells/100μL were seeded into ultralow attachment plates in serum-free mammary epithelium basal medium, supplemented with B27, EGF, insulin, ABM, and hydrocortisone. Cells grown in these conditions as non-adherent spherical clusters (mammospheres) were allowed to grow for 7 days. The mammosphere cell numbers and size were determined by Evos® transmitted light microscope (Life Technologies Co., Grand Island, NY). Duplicate measurment were performed for each experiment and the values represent mean of fold change ± SEM (*n* = 3). *; *p* < 0.05 compared to control

The massive increase in CYP1A1 expression in CSCs as evidenced by RT-PCR and immunofluorescence assays prompted us to evaluate the effects of AhR activation and subsequent CYP1A1 induction on the self-renewal capacity of breast CSCs. To address this point, we examined the effects of two well-known AhR agonists and potent CYP1A1 inducers, TCDD and DMBA, on mammosphere formation numbers as an indicator of increased or decreased in the self-renewal capacity of breast cancer cells. Figure 2 shows that treatment of MCF-7 cells with increasing concentration of TCDD (0.1, 1, and 10 nM) or DMBA (1.25, 2.5, and 5 μM) significantly increased mammosphere numbers by all tested concentrations. Captured images for mammospheres from MCF-7 cells treated with a single concentration of TCDD (10 nM) and DMBA (5 μM) were only shown.

### Effects of AhR/CYP1A1 activation and inhibition on breast CSCs marker, ALDH

**Fig. 3:**
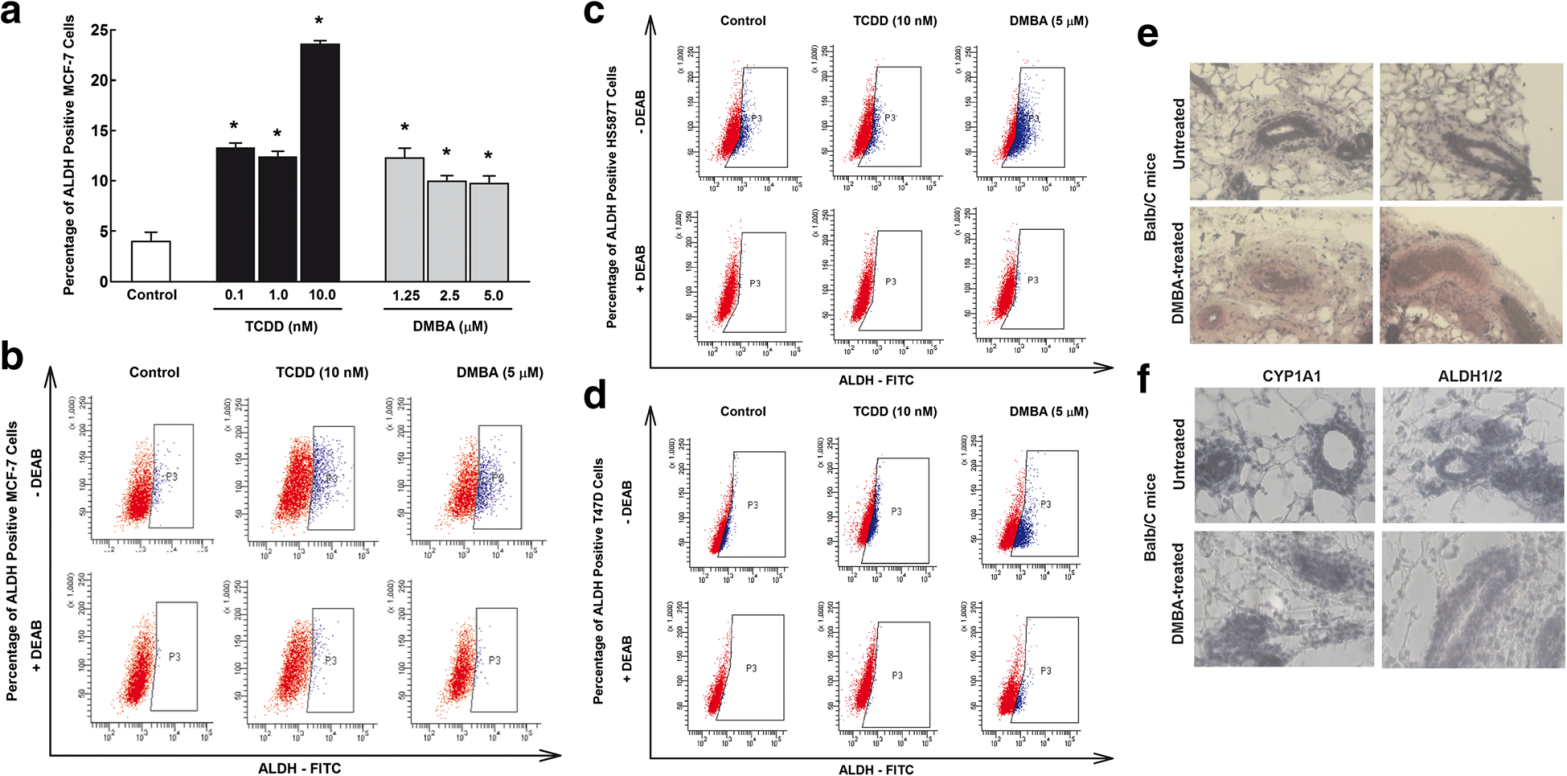
Effects of AhR/CYP1A1 activation on breast CSCs marker ALDH in vitro and in vivo. **a** MCF-7 cells were treated for 72 h with increasing concentrations of TCDD (0.1, 1, 10 nM) and DMBA (1.25, 2.5, and 5 μM). **b**-**d** Three human breast cancer cells; MCF-7, HS587T, and T47D cells were treated for 72 h with TCDD 10 nM and DMBA 5 μM. Pelleted cells were then incubated with ALDH substrate in the presence and absence of DEAB, ALDH inhibitor. Thereafter, percentage of ALDH+ cells were determined by flow cytometry. Duplicate reactions were performed for each experiment. **e**-**f** Twenty virgin female Balb/c mice were injected IP with either corn oil (vehicle) or single dose of DMBA 30 mg/kg. Two months later, mammary gland tissues were collected and stained with H/E for histopathology (**e**) or with antibody against CYP1A1 and ALDH1/2 for IHC assay (**f**)

The potential of AhR agonists and CYP1A1 inducers to increase self-renewal capacity was assessed using in vitro and in vivo mice models by measuring ALDH expression, a well-known specific marker for CSCs. At the in vitro level, MCF-7 cells were treated with increasing concentrations of TCDD (0.1, 1, and 10 nM) or DMBA (1.25, 2.5, and 5 μM), thereafter the percentage of ALDH positive cells was determined by Aldefluor assay using flow cytometry. Figure 3a shows that both AhR agonists TCDD and DMBA significantly increased the percentage of ALDH positive MCF-7 cells by all concentrations tested (Fig. 3a). In that, TCDD 10 nM and DMBA 5 μM significantly increased ALDH positive population of MCF-7 cells by approximately 5.75- and 2.5-fold, respectively (Fig. 3b). To further confirm the potential of TCDD and DMBA to increase ALDH activity of other cancer cell lines, breast cancer HS587T and T47D cells were incubated with TCDD 10 nM and DMBA 5 μM and the percentage of ALDH positive cells were determined. Fig. 3c shows that DMBA 5 μM significantly increased ALDH positive population of HS587T cells by approximately 2.3 -fold, whereas both TCDD 10 nM and DMBA 5 μM significantly increased ALDH activity of T47D cells by approximately 1.8- and 3-fold, respectively (Fig. 3d).

In order to evaluate the effect of AhR pathway modulation in vivo, we used DMBA-induced breast cancer model in Balb/c mice. For this purpose, virgin female Balb/c mice were treated with either corn oil (vehicle) or DMBA 30 mg/kg IP for 2 months. Thereafter, histopathological changes and the expression levels of CYP1A1 and CSC marker ALDH1/2 were determined in mammary gland structures by IHC. The histopathology examination (Fig. 3e) shows some proliferative tumor cells in a disorder patter which were bigger in size than normal epithelial cells. Furthermore, dense structure and necrosis were observed in the center associated with significant increase in vascular density. Importantly, immunohistochemical staining demonstrated a strong increase in the cytoplasmic and nuclear expression of CYP1A1 and the CSC marker ALDH in DMBA-treated mice mammary tissues (Fig. 3f). Taken together, these results strongly indicate the overexpression of CYP1A1 and CSCs marker ALDH in vivo and in vitro model.

**Fig. 4:**
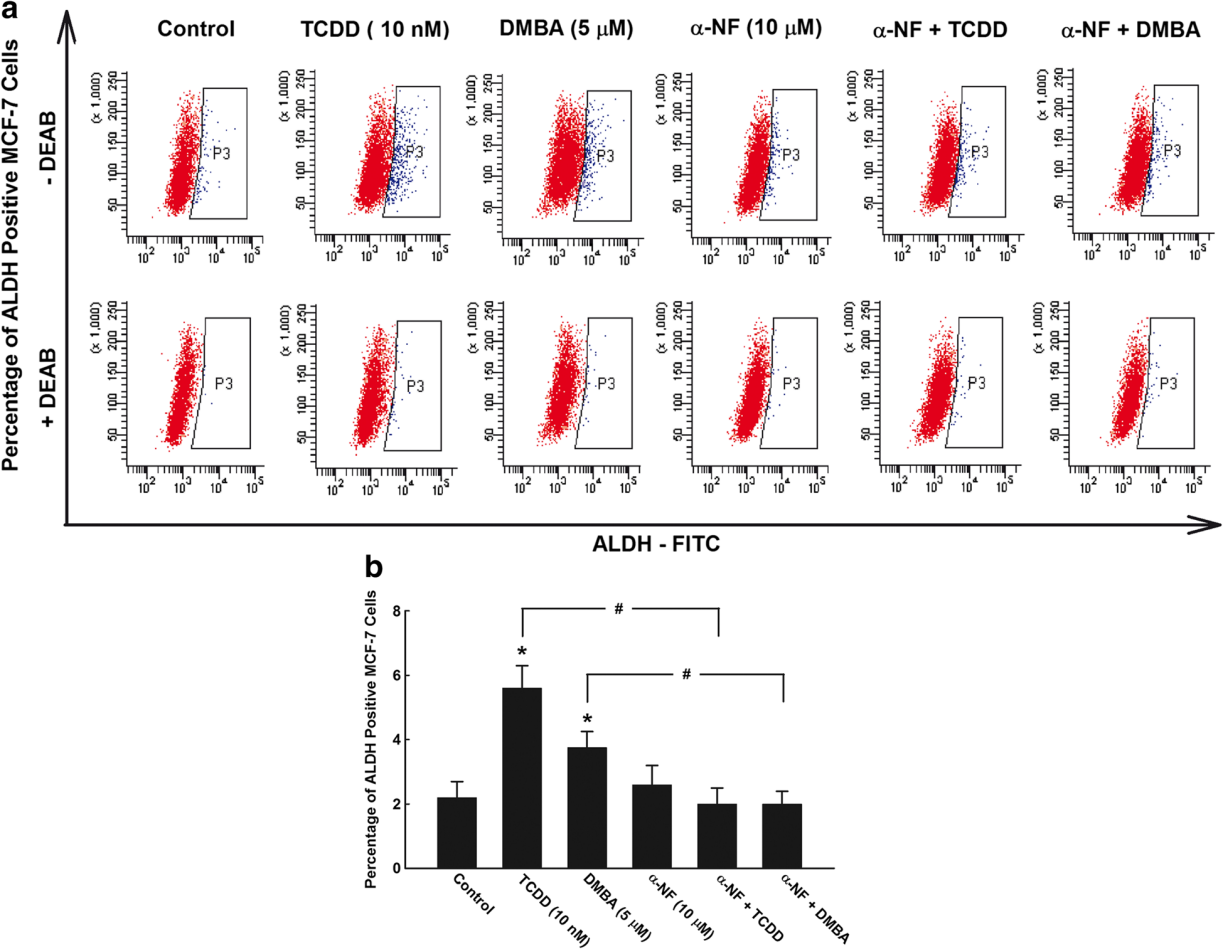
Effects of AhR/CYP1A1 inhibition on breast CSCs marker ALDH in vitro. **a** MCF-7 cells were treated for 72 h with TCDD 10 nM and DMBA 5 μM in the presence and absence of AhR/CYP1A1 inhibitor, α-NF 10 μM. Pelleted cells were then incubated with ALDH substrate in the presence and absence of DEAB, ALDH inhibitor. Thereafter, percentage of ALDH+ cells were determined by flow cytometry. **b** Duplicate reactions were performed for each experiment and the values represent mean of fold change ± SEM (*n* = 6). *; *p* < 0.05 compared to control. #; *p* < 0.05 compared to corresponding treatment in the absence of α-NF

In order to confirm the dependence of increased CSC marker ALDH on AhR/CYP1A1 pathway, we tested whether blocking of AhR/CYP1A1 pathway in MCF-7 cells using alpha-naphthoflavone (α-NF), a well-known AhR/CYP1A1 antagonist [33], would inhibit ALDH activity. To address this question, MCF-7 cells were pretreated with α-NF 10 μM for 2 h in the presence and absence of TCDD 10 nM or DMBA 5 μM for additional 72 h, thereafter ALDH activity was determined. Figure 4a and b shows that treatment of MCF-7 cells with α-NF alone did not significantly alter the basal number of ALDH stained cells, as compared to control cells. However, the increased ALDH positive cells by AhR agonists, TCDD (155%) and DMBA (70%) was abrogated by pretreatment with α-NF, AhR antagonist, to their control values. These observations strongly indicate that CSC marker ALDH is dependent on AhR/CYP1A1 pathway (Fig. 4a and b).

### Effects of AhR/CYP1A1 activation and inhibition on breast CSCs marker, side population (SP)

**Fig. 5:**
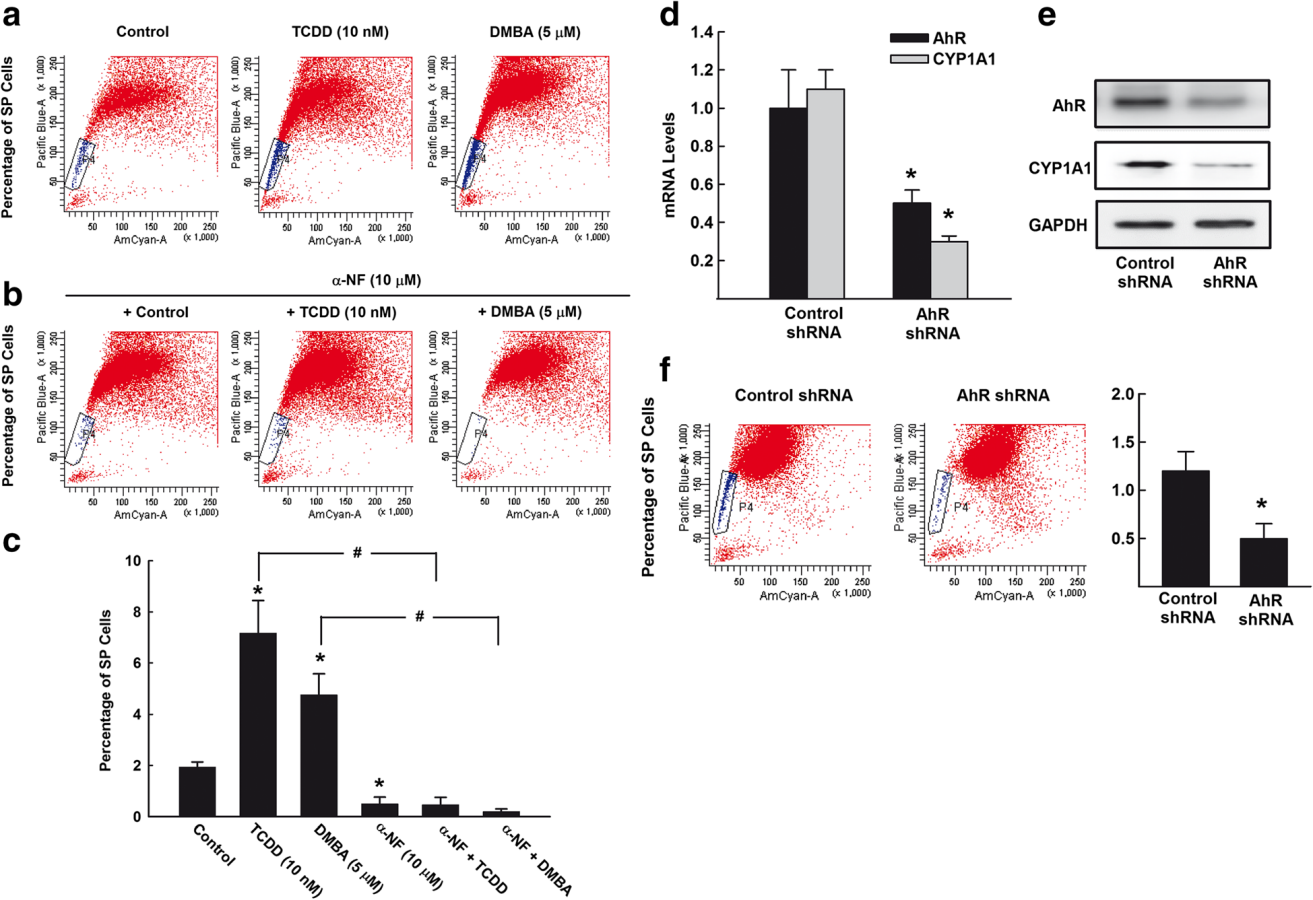
Effects of AhR/CYP1A1 activation and inhibition on breast CSCs marker SP. **a**-**c** MCF-7 cells were treated for 72 h with TCDD 10 nM and DMBA 5 μM (**a**) or in the presence of α-NF 10 μM (**b**). Pelleted MCF-7 cells were incubated with DCV (10 μM) and the percentage of SP cells were then determined using LSRII® flow cytometer cell sorter. **c** The values represent mean ± SEM (*n* = 3). *; *p* < 0.05 compared to control. #; *p* < 0.05 compared to corresponding treatment in the absence of α-NF. **d**-**f** MCF-7 cells were stably transfected with specific AhR shRNA, thereafter, (**d**) the mRNA expression of AhR and CYP1A1 were quantified by RT-PCR normalized to β-ACTIN housekeeping gene. Duplicate reactions were performed for each experiment and the values are presented as mean ± SEM (*n* = 6). *p < 0.05 compared to corresponding control shRNA. **e** AhR and CYP1A1 protein expression levels were determined by Western blot analysis using the enhanced chemiluminescence method and one of three representative experiments is shown. **f** Pelleted MCF-7 cells were incubated with DCV (10 μM) and the percentage of SP cells were then determined using LSRII® flow cytometer cell sorter. The values represent mean ± SEM (*n* = 3). *; *p* < 0.05 compared to control shRNA

Side population (SP) cells, as a marker for CSCs, are identified according to their ability to efflux the fluorescence dye at a higher pace than the remaining tumor cells (non-SP), which appeared as a distinct dim ‘tail’ in the flow cytometry plot. To examine the effect of AhR/CYP1A1 pathway on the SP, MCF-7 cells were treated with TCDD 10 nM and DMBA 5 μM, thereafter the percentage of SP cells was determined. Figure 5a shows that treatment of MCF-7 cells with either TCDD or DMBA resulted in an increase in SP of MCF-7 cells to 6.5% (3.2-fold) and 4.6% (2.3-fold), respectively, as compared to control (2%) (Fig. 5a and c).

Importantly, the direct evidence for involvement of AhR/CYP1A1 in SP cells was assessed by two approaches. First, we examined whether the AhR antagonist, α-NF, treatment would block SP cells. Figure 5b and c shows that pretreatment of MCF-7 ells with the AhR antagonist α-NF (10 μM) alone significantly inhibited the basal level of SP cells to 0.5% as compared to control (2%) (Fig. 5c). Interestingly, the increases in the percentage of SP cells by AhR agonists, TCDD (6.5%) and DMBA (4.5%) were markedly blocked by α-NF treatment to approximately less than 0.5% (Fig. 5b and c). The second approach to further confirm the role of AhR/CYP1A1 was by knockdown AhR using specific shRNA. Initially, we validated that knockdown of AhR resulted in a significant decrease in AhR and CYP1A1 mRNA expression levels by approximately 50% and 75%, respectively (Fig. 5d) which was associated with a proportional inhibition of AhR and CYP1A1 protein levels by approximately 50% and 85%, respectively (Fig. 5e). The most interesting result is the forced deceased of gene expression of both AhR and CYP1A1 resulted in a significant inhibition in SP cells by 50% as compared to control shRNA (Fig. 5f). Taken together, these results clearly demonstrate the involvement of AhR in controlling SP.

### Effects of AhR/CYP1A1 inhibition on CSCs chemoresistance

**Fig. 6:**
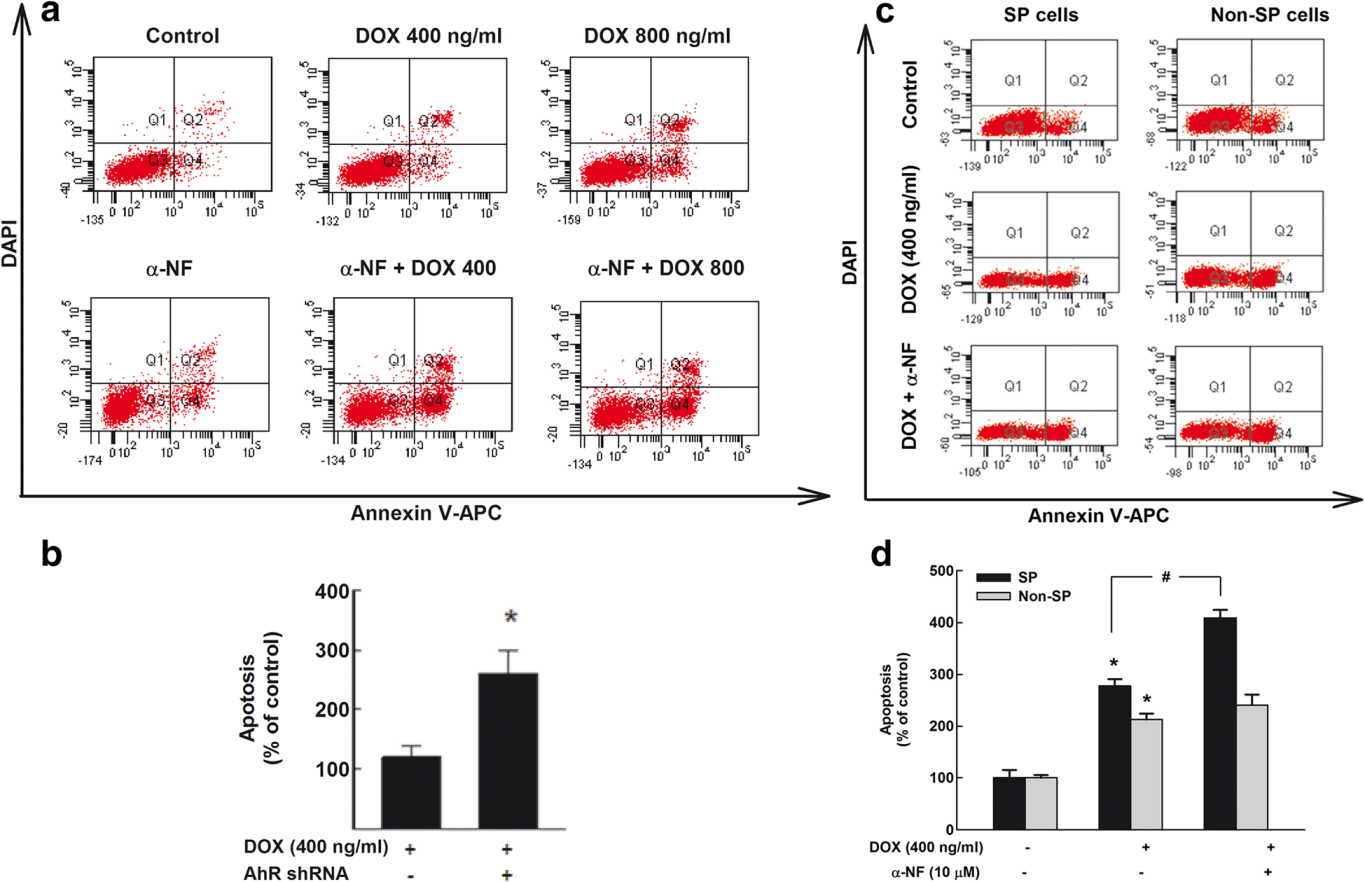
Effect of AhR inhibition on CSC apoptosis. **a** and **b** MCF-7 cells were treated for 72 h with DOX 400 or 800 ng/ml in the presence and absence of α-NF (10 μM) (**a**) or AhR shRNA (**b**). **c** and **d** SP and non-SP MCF-7 cells were treated for 72 h with DOX 400 ng/ml in the presence and absence of α-NF (10 μM). The cells were collected and then stained with annexin V–APC/DAPI. The percentage of cells underwent to apoptosis was then analysed on LSRII Flow Cytometer. One of the three representative experiments using different cell preparations was only shown. The values represent mean ± SEM (*n* = 3). *; *p* < 0.05 compared to control. #; *p* < 0.05 compared to DOX treatment

To investigate whether inactivation of AhR/CYP1A1 pathway will sensitize breast CSCs to chemotherapy, we tested the effect of α-NF treatment or AhR shRNA on the chemotherapeutic agent doxorubicin (DOX)-induced apoptosis. For this purpose, MCF-7 cells were treated with DOX 400 and 800 ng/ml in the presence and absence of α-NF 10 μM, thereafter the percentage of cells underwent apoptosis, as an indicator for chemoresistance, was determined by flow cytometry. Figure 6a shows that both doses of DOX 400 and 800 ng/ml increased the percentage of apoptotic cells as compared to control cells. Importantly, treatment with α-NF alone increased the percentage of apoptotic cells and further potentiated the apoptotic effect of DOX. Similarly, knockdown of AhR using AhR shRNA increased the percentage of apoptotic cells induced by DOX treatment by 150% (Fig. 6b).

To address the question of whether the apoptotic effect of α-NF is specific to CSCs, SP (CSCs) and non-SP MCF-7 cells were treated with DOX 400 ng/ml in the presence and absence of α-NF. Thereafter, the percentage of cells underwent apoptosis as indicator of chemotherapy sensitivity was determined by flow cytometry. Figure 6c and d shows that DOX significantly induced apoptosis in both SP and non-SP cells by approximately 2.7- and 2.1-fold, respectively. Importantly, co-treatment of α-NF with DOX further increased the apoptosis percentage in only SP to about 4-fold, but not non-SP cells.

### Molecular mechanisms controlling CSCs by AhR/CYP1A1 pathway

To explore the molecular events associated with AhR/CYP1A1-mediated effects on CSCs, the role of Wnt and Notch pathways, the most important self-renewal pathways in breast cancer, were investigated. Therefore, several independent experiments were conducted as follows:

### Role of Wnt and Notch signaling pathways in AhR/CYP1A1-mediated effects on CSCs

**Fig. 7:**
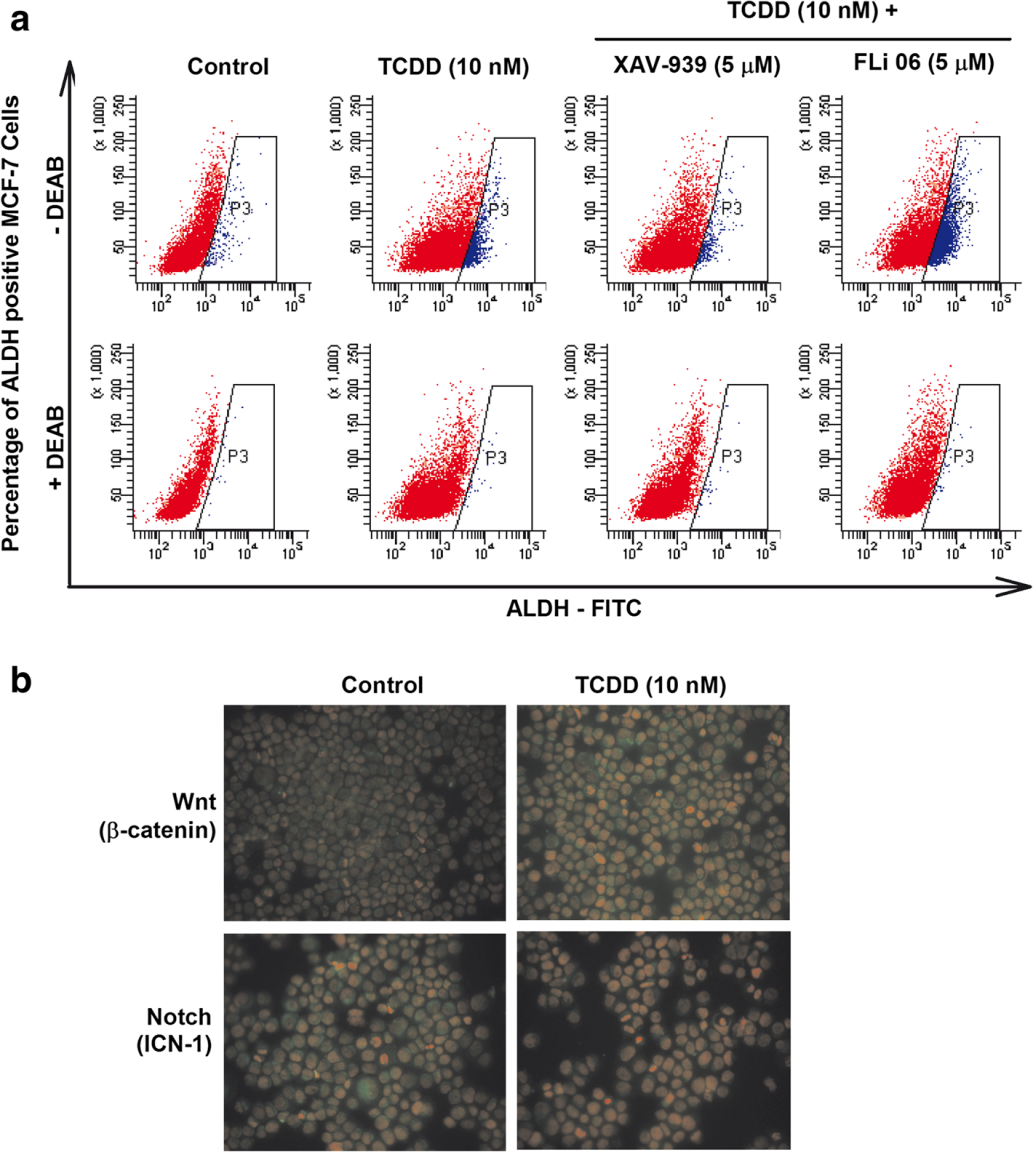
Effect of Wnt and Notch pathways inhibitors on TCDD-increased ALDH. **a** MCF-7 cells were treated for 72 h with TCDD 10 nM in the presence and absence of Wnt inhibitor (XAV-939, 5 μM) or Notch inhibitor (FLi 06, 5 μM). MCF-7 cell were then harvested, pelleted, and then incubated with ALDH substrate in the presence and absence of DEAB, ALDH inhibitor. Thereafter, percentage of ALDH+ cells were determined by flow cytometry. **b** MCF-7 cells treated with TCDD 10 nM were stained with primary antibodies against β-Catenin, Wnt transcription factor and ICN-1, Notch transcription factor followed by secondary antibodies. Thereafter, β-Catenin and ICN-1 proteins localization was conducted by immunofloursene assay. Each sample was stained in triplicate for each antibody

To determine whether AhR/CYP1A1 effects on CSCs development, maintenance and self-renewal are mediated through Wnt and/or Notch signaling pathways, MCF-7 were treated with TCDD 10 nM in the presence and absence of XAV-939 (5 μM), a selective inhibitor of Wnt/β-Catenin signaling pathway [34], or FLi 06 (5 μM), a Notch signaling pathway inhibitor [35]. Thereafter, ALDH + stained MCF-7 cells were determined using Aldeflour assay. Figure 7a shows that the TCDD-induced ALDH positive cells (10%), as compared to control (2.4%), was significantly inhibited by the Wnt transcription factor inhibitor, XAV-939, by approximately 65%. In contrast, Notch inhibitor, FLi 06, further increased ALDH positive cells by 2-fold. The involvement of Wnt, but not Notch, pathway was further confirmed by immunofluorescence assay by staining MCF-7 cells treated with TCDD 10 nM with antibodies against β-Catenin, the Wnt transcription factor, or intracellular notch 1 (ICN-1), a Notch transcription factor. Figure 7b shows that TCDD increased the expression and nuclear translocation of β-Catenin (green), whereas did not significantly alter the basal expression or translocation of ICN-1. Taken together, these results clearly suggest that Wnt, but not Notch, pathway is mediating AhR/CYP1A1 effects on CSCs.

### Effect of AhR/CYP1A1 activation and knockdown on β-Catenin expression and nuclear translocation

**Fig. 8:**
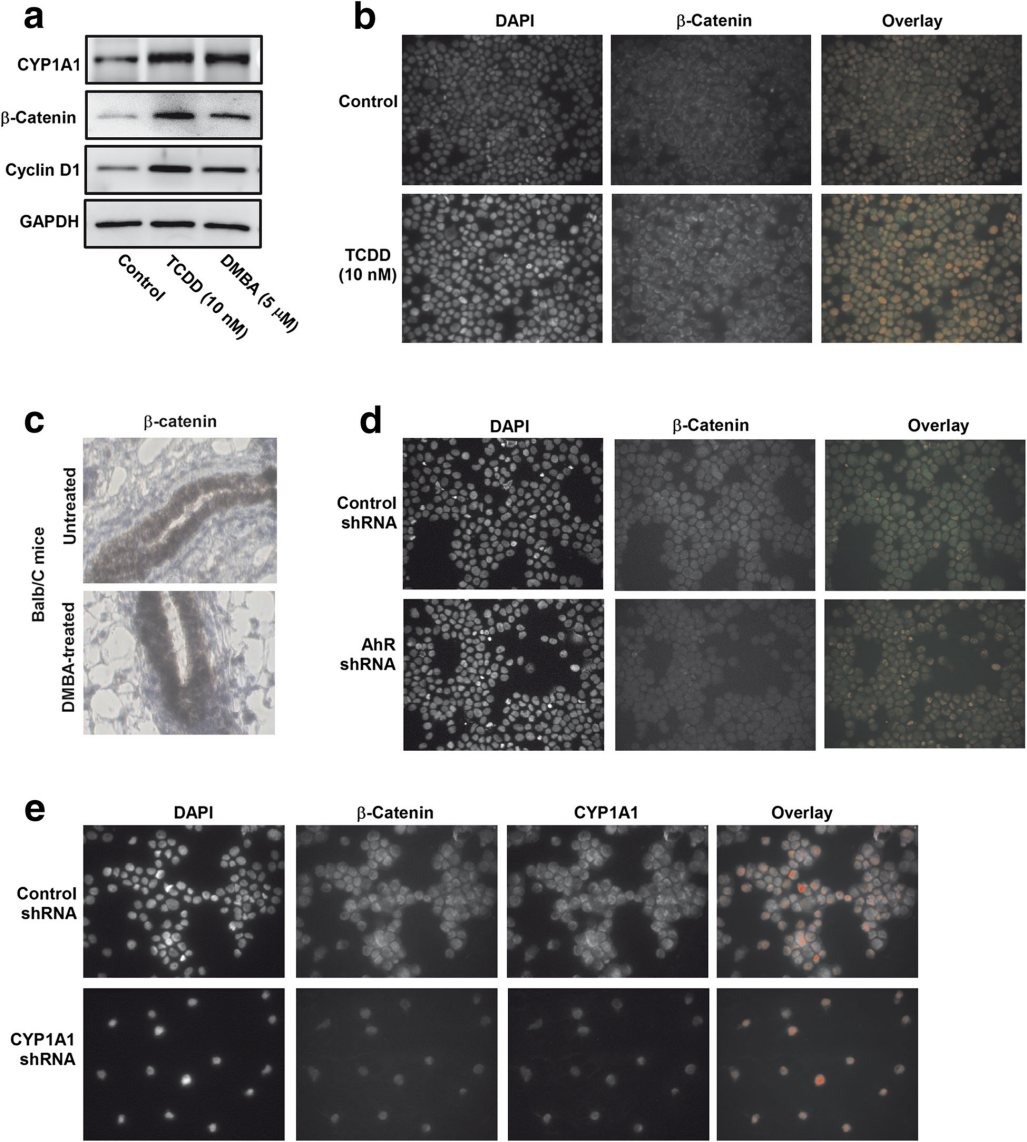
Effects of AhR/CYP1A1 activation and knockdown on β-Catenin levels in vitro and in vivo. **a** MCF-7 cells were treated with TCDD 10 nM and DMBA 5 μM for 72 h, thereafter the expression CYP1A1, β-Catenin, Cyclin D1 protein levels were determined by Western blot analysis using the enhanced chemiluminescence method and one of three representative experiments is shown. **b** MCF-7 cells treated with TCDD 10 nM were stained with primary antibodies against β-Catenin (green) followed by secondary antibodies and DAPI (red). Thereafter, β-Catenin localization and nuclear translocation was determined by immunofluorescence assay. Each sample was stained in triplicate for each antibody. **c** Twenty virgin female Balb/c mice were injected IP with either corn oil (vehicle) or single dose of DMBA 30 mg/kg. Two months later, mammary gland tissues were collected and stained with antibody against β-Catenin for IHC assay. **d** MCF-7 cells stably transfected with AhR shRNA (**d**) or CYP1A1 shRNA (**e**) were stained with primary antibodies against β-Catenin (green) and CYP1A1 (magenta) followed by secondary antibodies and DAPI (red). The β-Catenin and CYP1A1 localization and nuclear translocation were determined by immunofluorescence assay

The direct role of Wnt/β-Catenin pathway in AhR/CYP1A1 mediated effects on CSCs was assessed by two approaches. First, we examined the effect of AhR/CYP1A1 activation on the protein expression level of β-Catenin, a Wnt transcription factor, at the in vitro and in vivo levels. Using in vitro MCF-7 cells model, Western blot analysis (Fig. 8a) shows that both TCDD 10 nM and DMBA 5 μM induced CYP1A1 protein expression levels. Importantly, the CYP1A1 induction in response to TCDD and DMBA was associated with significant increases in the protein expression levels of β-Catenin and Cyclin D1 (β-Catenin downstream target [36]) which was more pronounced by TCDD than by DMBA. In addition, immunofluorescence assay (Fig. 8b) shows that staining of MCF-7 cells, treated with TCDD 10 nM, with antibody against β-Catenin showed a massive increase in β-Catenin cellular content and nuclear translocation as compared to control untreated cells (Fig. 8b). Similarly, immunohistochemical staining of mammary gland tissues of Balb/c mice treated with DMBA showed increased the expression and content of β-Catenin (Fig. 8c).

Second, we investigate the effect of AhR and CYP1A1 knockdown on the expression and nuclear translocation of β-Catenin using Immunofluorescence assay. Figure 8d and e shows that knockdown of the AhR using shRNA plasmid DNA gene silencing resulted in a decrease in β-Catenin (green) expression and nuclear translocation as compared to control shRNA (Fig. 8d), whereas, CYP1A1 shRNA completely inhibited CYP1A1 (magenta) and β-Catenin (green) expression and translocation (Fig. 8e).

### Role of PTEN/Akt Pathway in AhR/CYP1A1 mediated effects on CSCs

**Fig. 9:**
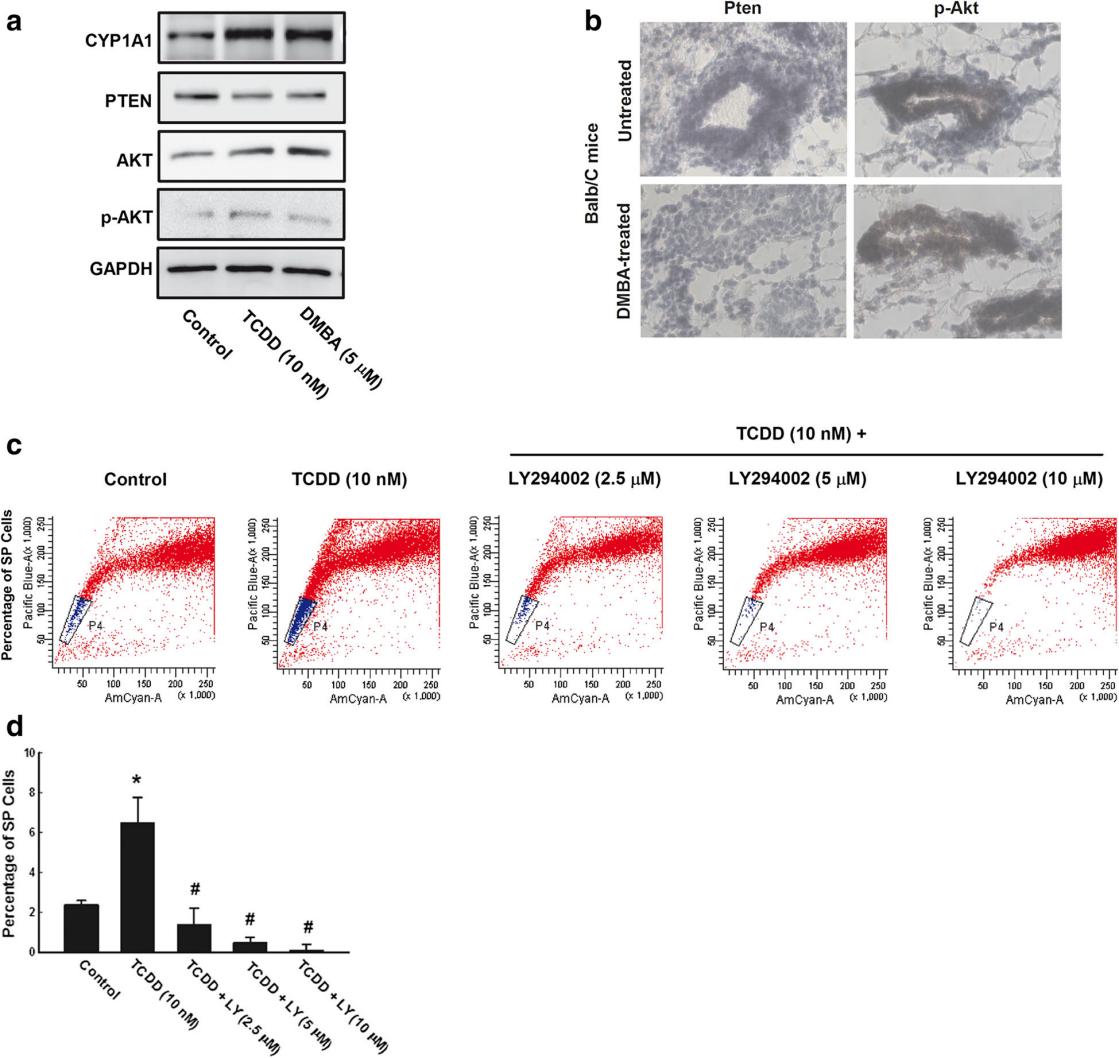
Effect of AhR activation on PTEN/Akt pathway in vitro and in vivo. **a** MCF-7 cells were treated with TCDD 10 nM and DMBA 5 μM for 72 h, thereafter the expression CYP1A1, PTEN, Akt, and p-Akt protein levels were determined by Western blot analysis using the enhanced chemiluminescence method and one of three representative experiments is shown. **b** Twenty virgin female Balb/C mice were injected IP with either corn oil (vehicle) or single dose of DMBA 30 mg/kg. Two months later, mammary gland tissues were collected and stained with antibody against PTEN and p-Akt for determination by IHC assay. **c** and **d** MCF-7 cells were treated for 72 h with TCDD 10 nM in the presence and absence of increasing concentrations of Akt pathway inhibitor, LY294002. Thereafter, cells were harvested, pelleted, and then incubated with DCV (10 μM) and the percentage of SP cells were then determined using LSRII® flow cytometer cell sorter. The values represent mean ± SEM (*n* = 3). *; *p* < 0.05 compared to control, #; *p* < 0.05 compared to TCDD treatment

Several studies have demonstrated a cooperation between Wnt/β-Catenin and PTEN-PI3K/Akt pathway in controlling CSCs self-renewal and expansion [37–39]. To test whether AhR/CYP1A1 effect on CSCs is mediated by PTEN/Akt pathway, we first examined the effect of AhR activation by TCDD and DMBA on the expression levels of several PI3K regulated proteins, Akt, phosphorylated Akt (p-Akt), and PTEN in vitro MCF-7 cells and in vivo Balb/c mice models. Figure 9a shows that induction of CYP1A1 protein level in MCF-7 cells by TCDD and DMBA treatment was associated with significant loss of PTEN protein level and activation of Ak and p-Akt protein expression levels. Such effects were more pronounced by TCDD than DMBA (Fig. 9a). Similarly, treatment of female Balb/c mice with DMBA for 2 months decreased PTEN and increased p-Akt staining using IHC (Fig. 9b).

Second, we tested whether blocking of Akt pathway by LY294002, a specific inhibitor of PI3 kinase-dependent Akt phosphorylation and kinase activity, would block the capacity of TCDD to increase CSCs marker, SP. For this purpose, MCF-7 cells were treated with TCDD 10 nM in the presence and absence of increasing concentrations of LY294002 (2.5, 5, and 10 μM), thereafter percentage of SP cells was determined. Figure 9c and d shows that the TCDD-increased MCF-7 SP cell percentage (6.5%) as compared to control (2.2%) was completely abrogated by LY294002 treatment in a concentration-dependent manner to approximately, 1.2%, 0.5%, and 0.1%, respectively. These results indicate that AhR/CYP-mediated CSCs self-renewal is dependent on PTEN-PI3K/Akt pathway.

## Discussion

Epidemiological studies have shown that exposure to environmental pollutants such as AhR agonists (TCDD) results in a significant increase in the incidence and pathogenesis of lymphoma and leukemia [40]. In addition, a highly significant correlation between the AhR expression and invasive breast carcinoma was reported [41]. Recent studies have demonstrated that activation of AhR dramatically increased CSCs development and characteristics [21, 42]. D’Amato and coworkers have recently demonstrated that Kynurenine substance generated by TNBC activates AhR causing the receptors to translocate to the cell’s nucleus to regulate the expression of target genes involved in cancer cell migration and immune system suppression [43]. Although part of the results of the current study are in agreement with Stanford et al. [40], these studies lack the mechanistic investigation of the role of Wnt/β-Catenin and PTEN/Akt pathways on AhR/CYP1 effects on CSCs. In contrast, previous studies have reported a protective effect of AhR agonist on CSCs of TNBC [44] and MCF-7 breast cancer cells via downregulation of Wnt/β-Catenin and Notch signaling pathway [45]. These discrepancies warranted further investigations to explore the role and molecular mechanism of the AhR and regulated genes in developing, maintaining, and generating chemotherapy-resistant CSCs, which are fully uninvestigated. Therefore, the current study was designed to examine the functional relevance of constitutive AhR/CYP1A1 expression, AhR/CYP1A1 activation by two environmental carcinogens TCDD and DMBA, and AhR/CYP1A1 inhibition using chemical inhibitor and gene silencing on breast CSCs and to explore the molecular mechanisms involved particularly Wnt/β-Catenin, Notch/TCF, and PI3K/Akt signaling pathways.

To test our hypothesis, we initially investigated the constitutive expression of AhR and its regulated genes, CYP1A1 and CYP1B1 in different breast cancer cell lines. At the constitutive level, five breast cancer cell lines that exhibit different hormonal heterogenicity, particularly SKBR-3 (HER2+), MCF-7 and T47D (ER+), and HS587T and MD-MB 231 (TNBC), were utilized to explore the differential constitutive expression of AhR-regulated genes, CYP1A1 and CYP1B1 in the mammospheres (CSCs) vs adherent (non-CSCs) cells. The results of these experiments have demonstrated for the first time that the basal expressions of CYP1A1 and CYP1B1 mRNA levels were markedly overexpressed in mammosphere more than in adherent cells of all tested breast cancer cells lines, among which, MCF-7 mammosphere cells showed the highest expression levels of CYP1A1 and CYP1B1 followed by T47D cells. In addition, the CYP1A1 content and cellular localization were higher in MCF-7 mammosphere populations than adherent non-CSCs. Importantly, the increased CYP1A1 expression and cellular content were accompanied with a proportional increase in the cellular levels of ALDH, one of the most identifying marker for CSCs.

At the inducible level, two AhR agonists and CYP1A1 inducers, TCDD and DMBA, have been utilized to examine the effect of AhR/CYP1A1 on CSCs properties and development. TCDD is classified as a known human carcinogen and potent tumor promoter, whereas, DMBA is a powerful organ-specific laboratory carcinogen and tumor initiator that is widely used as a chemical carcinogen in rat mammary tumor model [46, 47]. The carcinogenicity and tumoreginicity of TCDD and DMBA are attributed to the ability to induce CYP1A1 and CYP1B1. In the context of CYP1B1 carcinogenicity, a recent study on breast CSCs have demonstrated that CYP1B1 mRNA expression was significantly increased in ALDH positive HS578T (TNBC) cells and this increase was associated with increased migration/invation genes [40]. In the present study, activation of AhR/CYP1A1 pathway by TCDD and DMBA resulted in increased CSCs featured and markers. In that, both TCDD and DMBA increased the mammosphere formation numbers in MCF-7 cells, the ALDH+ activity in MCF-7, HS587T, and T47D cells, and the percentage of SP in MCF-7 cells. Similarly at the in vivo level, DMBA treatment of female Balb/c mice for 2 months resulted in a significant histopathological changes associated with strong increase in the cellular distribution and content of CYP1A1 and CSC marker, ALDH. These results indicate that AhR/CYP1A1 activation increases self-renewal capacity of CSCs as reflected by the increase in population for CSCs marker, ALDH and SP. In this context, it was reported that increased the degree of efflux activity of CSCs (SP) correlates highly with the maturation state and differentiation potential. In addition, a high ALDH activity of breast carcinoma cells can enrich tumorigenic stem/progenitor cells, in which only 500 ALDH+ cells can induce tumor formation that are resistant to conventional chemotherapy [10]. In agreement with our results, Jung and co-workers have demonstrated the ability of TCDD to increase MCF-7 cell proliferation and the number and size of the mammospheres [21].

The role of AhR/CYP1A1 pathways in controlling CSCs development and self-renewal was addressed by two approaches. First investigating whether inhibition of AhR/CYP1A1 pathway using chemical inhibitor, α-NF, would block the TCDD-increased ALDH+ and SP cells. The AhR antagonist, α-NF, significantly decreased the TCDD- and DMBA-mediated increased percentage of ALDH+ and SP cells. Since α-NF acts as an AhR antagonist in MCF-7 cells by decreasing the levels of transcriptionally active nuclear AhR complexes with very low affinity for DNA [33, 48], the increased ALDH+ cells as a CSCs is thought to be mediated through an AhR/CYP1A1-dependent and metabolism-independent mechanism. The results derived from the chemical inhibitor study were further confirmed by AhR shRNA knockdown study which showed that silencing of AhR or CYP1A1 significantly decreased the percentage of SP cells as compared to control shRNA. Taken together, these results strongly indicate that modulation of AhR/CYP1A1 pathway would have a significant impact on controlling CSCs proliferation, maintenance, and self-renewal. Perhaps the most interesting results in the current study are the observations that inhibition of the AhR/CYP1A1 by α-NF further sensitized CSCs to chemotherapy as evidenced by increased the percentage of apoptotic SP (CSCs) in response to DOX more than in non-SP cells. These results suggest, at least in part, the involvement of AhR/CYP1A1 in CSCs chemoresistance.

There are several signaling pathways that have been demonstrated to contribute to CSCs development and maintenance and thus are critically important in CSCs biology. Among these pathways, Wnt pathway through β-Catenin/TCF transcription factor is known to mediate CSC self-renewal, whereas CSCs maintenance and differentiation are controlled by Notch/Hes pathway [12]. However, whether Wnt and/or Notch signaling pathways mediate AhR/CYP1A1 effects on CSCs properties remains unclear. In this context, we reported here that Wnt signaling pathway, but not Notch, is controlling AhR/CYP1A1 effects. This conclusion is supported by three observations, first blocking of the Wnt pathways using specific inhibitor, XAV-939, significantly inhibited the increase of ALDH+ cells by TCDD, whereas Notch pathway inhibitor, FLi 06, further increased SP cells. Second, treatment with TCDD significantly increased the nuclear localization of β-Catenin, Wnt transcription factor, without any significant changes in the Notch transcription factor, ICN-1. Third, induction of CYP1A1 protein expression level by TCDD and DMBA was accompanied with a proportional increase in the protein expression levels of β-Catenin and its downstream protein, Cyclin D1. Importantly, these in vitro observations were consistent with in vivo mice study, in which DMBA treatment for 2 months significantly increased the cytoplasmic and nuclear expression of β-Catenin as evidenced by IHC assay. All collectively suggest that Wnt/β-Catenin pathway likely plays an important regulatory role in AhR-mediated activation of CSCs. This possibility is supported by the observations of a recent study reported a cross talk between the Wnt pathway and AhR pathway.

Wnt/β-Catenin pathway is known to play key roles in expansion of stem and/or progenitor cell in several tissues, in that studies on mouse and human breast cancer models have revealed that Wnt signaling is critical to mammary tumorigenesis. In the current study, the role of Wnt/β-Catenin pathway was further confirmed by investigating whether silencing of AhR and CYP1A1 gene expressions via small hairpin RNA (shRNA) would decreased β-Catenin cellular level and nuclear localization. In this context, we reported here that AhR knockdown decreased the nuclear and cellular localization of β-Catenin as compared to control shRNA, whereas silencing of CYP1A1 completely inhibited β-Catenin levels. These observations not only indicate that AhR/CYP1A1 controls β-Catenin activations, but also suggest a possible cross talk between AhR/CYP and Wnt/β-Catenin pathways. In this regards, mechanistic studies have demonstrated a physical interaction between AhR and β-Catenin, in that activation of β-Catenin enhances AhR activity at its DNA responsive element [49].

Studies on CSCs have demonstrated that self-renewal is cooperatively controlled and promoted by Wnt/β-Catenin and PTEN-PI3K/Akt pathways, in which neither pathway alone is sufficient to promote self-renewal. It has been recently reported that PTEN is critical for CSCs maintenance, in that PTEN loss results in activation of Akt with subsequent development of CSCs and ultimately tumorigenesis [50]. Thus, we questioned whether PTEN-PI3K/Akt pathway is mediating TCDD/DMBA effect on CSCs. We reported here for the first time that induction of CYP1A1 by TCDD or DMBA significantly downregulated the protein expression of PTEN associated with activation of Akt and its phosphorylated proteins in vitro MCF-7 cells and in vivo Balb/c mice model. Impotently, inhibition of Akt pathway by LY294002 completely (90%) blocked the TCDD-increased SP in a concentration-dependent manner, suggesting that PTEN-PI3K/Akt activity is essential component for SP cells phenotype regulation by AhR/CYP1A1 pathway.

## Conclusions

The present study provides the first evidence that AhR/CYP1A1 signaling pathway is controlling human breast CSCs development, maintenance, and self-renewal through β-Catenin and PTEN/Akt mechanisms. The results of the present study are of a great relevance to AhR/CSC field, as it provides a better understanding of the regulation of CSCs that may lead to more effective cancer prevention and therapy.

## Abbreviations

AhR: Aryl hydrocarbon receptor
ALDH: Aldehyde dehydrogenase-1
CSCs: Cancer stem cells
CYP1A1: Cytochrome P450 1A1
DMBA: 7,12-dimethybenz[a]anthracene
SP: Side population
TCDD: 2,3,7,8-tetrachlorodibenzo[p]dioxin
α-NF: Alpha-naphthoflavone
