# Erratum to: Aryl hydrocarbon receptor/cytochrome P450 1A1 pathway mediates breast cancer stem cells expansion through PTEN inhibition and β-Catenin and Akt activation

**DOI:** 10.1186/s12943-017-0636-5

**Published:** 2017-03-24

**Authors:** Abdullah Al-Dhfyan, Ali Alhoshani, Hesham M. Korashy

**Affiliations:** 1 Department of Pharmacology and Toxicology, College of Pharmacy, King Saud University, P.O. Box 2457, Riyadh, 11451 Saudi Arabia; 2 Stem Cell & Tissue Re-Engineering, King Faisal Specialist Hospital and Research Center, Riyadh, 11211 Saudi Arabia

## Erratum

After the publication of this work [1] an error was noticed in the legends for Figs. 5 and 9. The word ‘cell sorter’ should be deleted from the legend of Fig. 5 in line 3 and the last line. In the legend of Fig. 9 the word ‘cell sorter’ should be deleted from line 7.

The original article was corrected.
